# Fossilized Teeth as a New Robust and Reproducible Standard for Polarization-Sensitive Optical Coherence Tomography

**DOI:** 10.1155/2013/391972

**Published:** 2013-05-08

**Authors:** Christopher Vercollone, Bin Liu, Mark E. Brezinski

**Affiliations:** 1 Center for Optical Coherence Tomography and Optical Physics, Department of Orthopedic Surgery, Brigham and Women’s Hospital, 75 Francis Street, Boston, MA 02115, USA; 2 Harvard Medical School, Boston, MA 02115, USA

## Abstract

A clinical need exists for a cheap and efficient standard for polarization sensitive optical coherence tomography (PS-OCT). We utilize prehistoric fossilized teeth from the Megalodon shark and European horse as an unconventional, yet robust standard. Given their easy accessibility and the microstructural consistency conferred by the process of fossilization, they provide a means of calibration to reduce error from sources such as catheter bending and temperature changes. We tested the maximum difference in birefringence values in each tooth and found the fossilized teeth to be fast and repeatable. The results were compared to measurements from bovine meniscus, tendon, and destroyed tendon, which were verified with histology.

## 1. Introduction

Polarization-sensitive optical coherence tomography (PS-OCT) is a micron-scale imaging/spectroscopic modality capable of assessing organized structure in biological tissues through birefringence [1]. Day-to-day variation in the imaging systems limits the precision of repeated measurements, requiring the need for standards. The sources for variation are numerous, including the movement of fiber optic components within the system or catheter/endoscope, and are prominent in producing error [2, 3]. Preventing this variation by checking and recalibrating the system using a cheap, widely accessible standard would greatly increase the robustness of imaging. Reproducible standards have been difficult to achieve. In this paper, we have found an unusual standard, fossilized prehistoric marine vertebrate (predominately megalodon) and dinosaur teeth. Being at least 10’s of millions of years old, their microstructure and birefringence have been stabilized with time [4]. We compare their results to the OCT imaging of birefringent tendon and cartilage, validating the results with histopathology.

Optical coherence tomography is an imaging modality analogous to ultrasound, using low-coherence interferometry to measure the backreflection of infrared light rather than sound [5]. Already approved in both cardiology and ophthalmology for clinical imaging, OCT functions above video rate and at resolutions up to 25x higher than any other clinical imaging modality [6]. With respect to the single channel PS-OCT modality, as highly organized tissue is typically birefringent, it modifies the incident light polarization state, resulting in variations in the backreflection intensity. As early diseases progresses, birefringent materials such as collagen or enamel become disorganized, leading to a loss of birefringence. This occurs long before the gross structural changes identified by other imaging modalities [6, 7]. We have utilized single-detector/channel PS-OCT (rather than dual) in humans in vivo and in vitro for several reasons. These reasons include the fact physicians in general use categorical data with Bayesian-like processing, and also technical reasons, which are reviewed elsewhere and addressed in more detail in the discussion.

Many clinically relevant birefringent microstructures exist, including actin-myosin complexes, dentin, myelin, cholesterol crystals, and collagen [6]. Two areas of extensive PS-OCT investigation are the assessment of collagen in musculoskeletal tissue and in coronary arteries [6-9]. When collagen is organized in a normal, nonpathologic state, the tissue retains this component of birefringence and backscattering changes with depth. Conversely, disorganization of collagen is shown by loss of sensitivity to the light polarization state and can be indicative of early pathology. In musculoskeletal disease, for example, in osteoarthritis (OA), collagen disorganization represents early disease prior to cartilage thinning [8]. In cardiology, depletion of intima collagen is a major component in the destabilization of coronary plaques. Rupture of mechanically weak plaque is the major cause of myocardial infarction, or heart attacks [10, 11]. Loss of birefringence correlates with loss of collagen and instability.

PS-OCT systems control the polarization state of incident light through the mechanical manipulation of the fiber optics. Various embodiments exist, such as manual paddles, galvanometer driven paddles, and fiber squeezers. However, they are all susceptible to the generation of polarization error through fiber distortion. Even conditions such as temperature variation can cause minimal fiber distortion [2, 3, 6]. In addition, surface reflections and other variables produce even greater error. The ability to minimize this variation through simple calibration would greatly improve the significance of day-to-day measurements.

A reliable, inexpensive, easily obtainable standard for PS-OCT has not been published. As our group is using single detector OCT in vivo in humans, checking calibration of the polarization with filters and mirrors is impractical. There is a need for a reproducible standard that can give rapid confidence that the polarization settings of the system have not changed. We intend to introduce a birefringent standard that is easily obtainable and inexpensive, and which can be used as consistent birefringence standard for day-to-day system calibration. This work introduces, as a reliable but atypical standard, prehistoric fossilized teeth from the megalodon shark. As these birefringent teeth have been fossilized and remain unchanged over long periods of time, they prove to be very effective standards. They provide clean, easy samples to perform polarization calibration. In addition, we will be comparing the results of their polarization characteristics with those of bovine tendon and meniscus. The collagen organization of these samples is then confirmed with corresponding histological analysis. The paper introduces prehistoric teeth, particularly the megalodon, as a robust standard for PS-OCT.

## 2. Methods

### 2.1. Samples.

**Figure 1:**
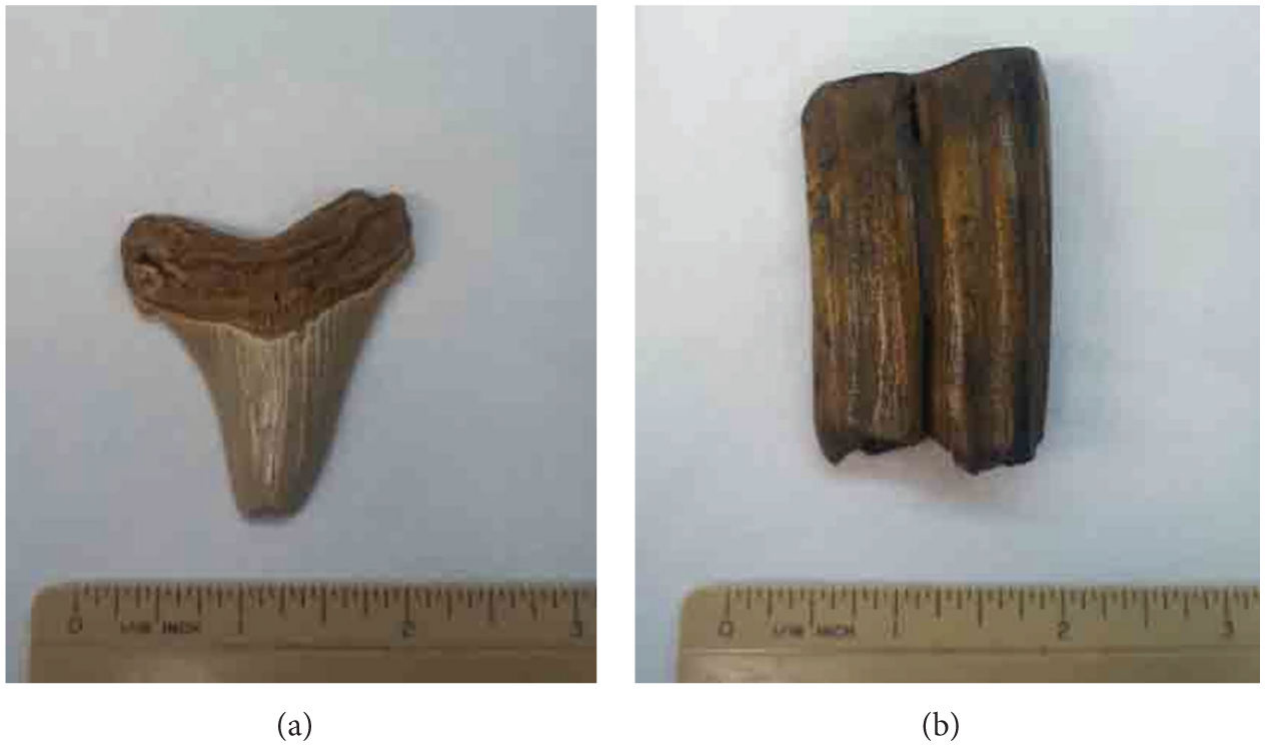
Pictures of fossilized standards. (a) Fossilized megalodon tooth. (b) Fossilized European horse tooth.

Fossilized samples were obtained from Paleo Direct Inc. (Almonte Springs, FL), including a prehistoric megalodon shark tooth and prehistoric European horse tooth (Figure 1). Bovine tendon and meniscus were also imaged to compare with histological analysis. Both tendon and meniscus had been previously fixed in 10% buffered formalin for 24 hours before being stored in saline. Half of the tendon sample was then physically crushed and treated with 1M HCl for 24 hrs to both chemically and physically destroy organization and composition properties.

### 2.2. OCT System.

**Figure 2:**
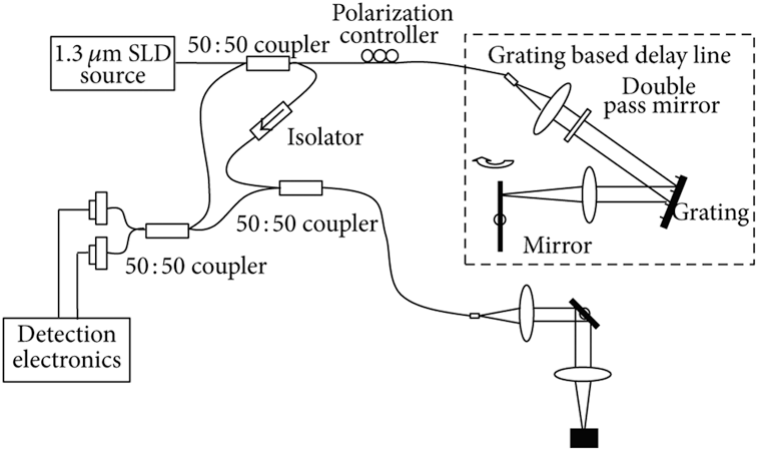
Schematics of single detector PS-OCT systemused in this study.

Imaging was performed with a modified Thorlabs Swept Source OCT System (Thorlabs, Newton, NJ, USA) (Figure 2). The system consists of a 10mW, 1325nm infrared tunable laser light source at 6mm coherence length, producing a 12 μm axial resolution and 15 μm transverse resolution. Imaging was performed at a 25 frame/second data acquisition rate. A 3-paddle external polarization controller was added to the reference arm. The first paddle rotation was controlled through a manual program designed in Hyper Terminal, with 0.9 degree rotational increments possible over a 260 degree range. The other two paddles were at fixed angles. Room temperature was kept constant during the duration of the study. No other work was done with the system in order to prevent movement of fiber optic components that could alter polarization.

### 2.3. Imaging Protocol.

For each sample, a spot was imaged at 5 different time points. The edges of imaging planes were marked with microapplication of dye and samples were mounted in a constant position to guarantee imaging of the same planes at different time points. At each time point, the second and third paddles of the polarization controller were placed at fixed positions of 90° and 180°. The first paddle again was electronically controlled to move in 0.9° increments. Based on our previous work on reference arm tailoring, this was a rotation of reference arm polarization through linear polarization states. For each sample, at each time point, the paddle was rotated to the point where backreflection intensity was at a distinct peak and a distinct trough. The difference between the peak and trough backscattering intensity during polarization rotation was measured.

### 2.4. Histology.

For tendon and meniscus, samples underwent histological processing, maintaining the imaging planes for sectioning. Sections were stained with both Masson’s Trichrome, to expose any structural abnormalities, and picrosirius red, to determine collagen content and organization.

## 3. Results

**Figure 3:**
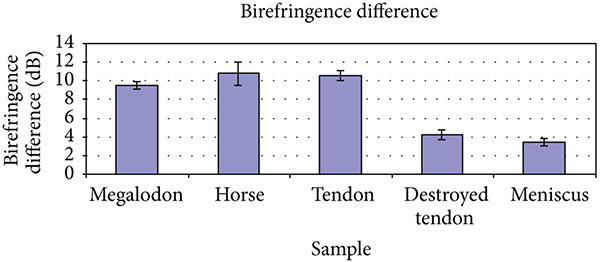
Difference between maximum and minimum backreflection intensity of peak as polarization paddle is rotated (in dB).

The difference in backreflection intensity for each sample is seen in Figure 3. As the polarization of the incident light was rotated, the degree of change in backreflection intensity was representative of the degree of birefringence. Three samples recorded values that were indicative of high birefringence. Both the prehistoric horse tooth and megalodon tooth were birefringent (values of 9.5 dB and 10.8 dB, resp.), and the bovine tendon was also highly birefringent (10.6 dB). Conversely, both the destroyed tendon and meniscus samples recorded lower birefringence values (4.2 dB and 3.4 dB, resp.).

**Figure 4:**
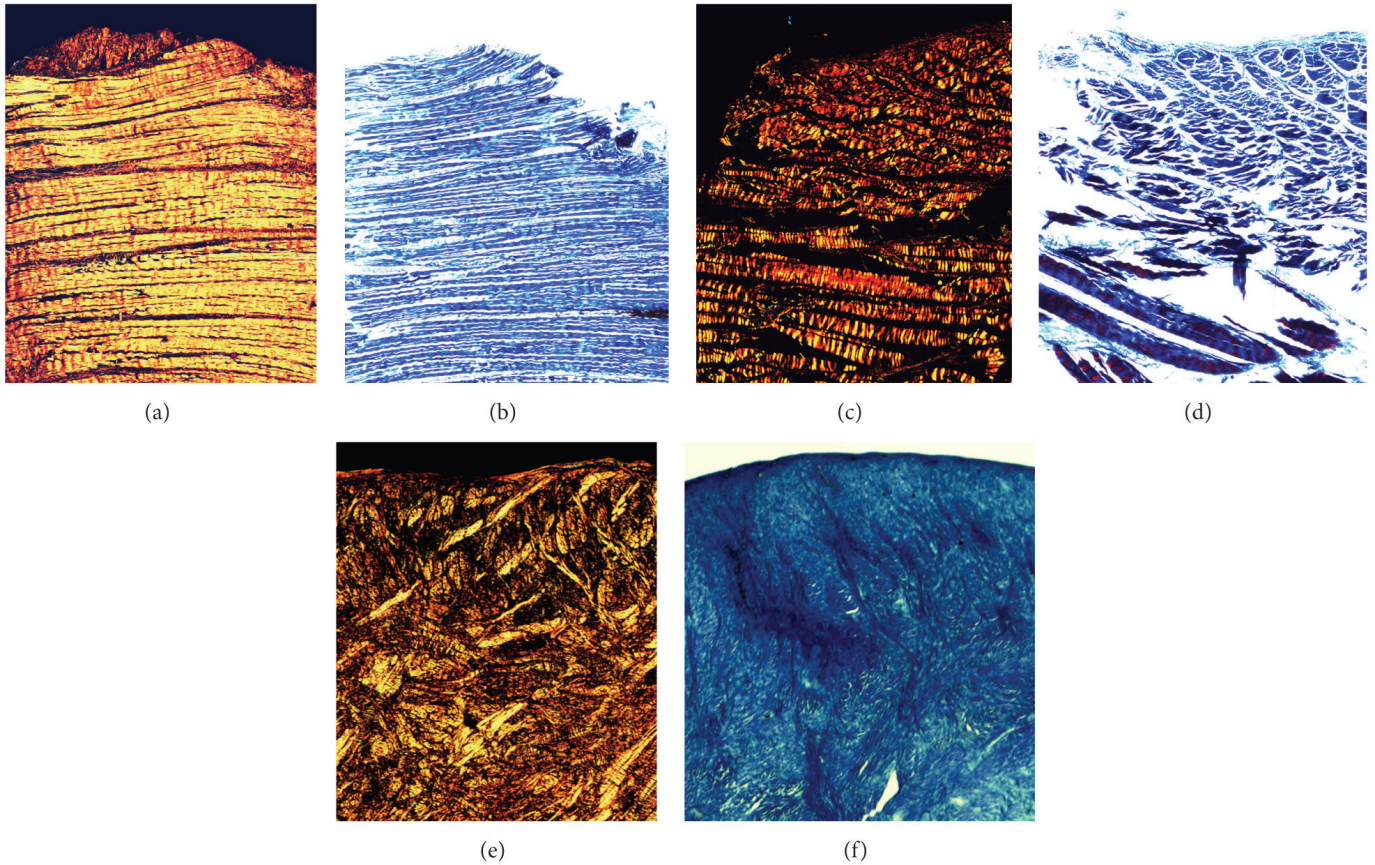
Histology of tendon and meniscus samples. (a) Picrosirius stained normal bovine tendon exhibits a very organized, bright yellow banding pattern indicative of high collagen content and good organization. (b) Trichrome of bovine tendon confirms structural robustness of tendon, again showing uniform bands. (c) Picrosirius staining of mechanically and chemically destroyed bovine tendon shows very little yellow banding and minimal organization, indicative of heavy collagen disruption and loss. (d) Trichrome of destroyed bovine tendon confirms picrosirius, showing structural rupturing. (e) Picrosirius staining of bovinemeniscus shows moderate lack of bright yellow banding, indicating disorganization and moderate collagen content. (f) Trichrome shows no structural abnormalities.

The birefringence of the tendon and meniscus samples was verified with histology (Figure 4). The normal tendon sample shows no structural abnormalities with Trichrome staining and bright, organized yellow banding with picrosirius staining. Both these stains indicate healthy, birefringent tissue. The meniscus sample shows no structural abnormalities via Trichrome staining, but shows moderately disorganized collagen structure with an overall moderate loss of collagen content. Although not severely structurally disrupted, birefringence is lost with the collagen organization and content loss. The destroyed tendon shows both structural degradation via Trichrome staining and severe collagen loss and disorganization via picrosirius staining. This indicates severe birefringence loss and microstructural degradation, consistent with the chemical and mechanical destruction.

## 4. Discussion

Variations in PS-OCT measurements can occur for several reasons, which will be discussed below. We have used PS-OCT extensively in the OR to assess collagen. The use of polarization filters to check the system before and between cases is impractical, so a robust standard which can be rapidly used is needed to allow confidence in the validity of measurements. A reliable standard that can be quickly used would not only be convenient, but also invaluable for preventing inaccurate data for any PS-OCT imaging in areas such as the OR and the cath lab. This paper supports megalodon teeth as such a standard.

In this study, our standard was tested on a single channel PS-OCT system. We prefer this particular embodiment for multiple reasons. For clinical use, the real-time relative measurements provided by single-detector PS-OCT allow quick interpretation of physician-preferred categorical data. In addition, the artifacts which these standards aim to correct are less numerous in the single-channel embodiment. The main artifacts generated by single-channel PS-OCT are distortions caused by temperature changes or alterations in the reference arm state. The latter is important, as we have recently demonstrated that single detector PS-OCT has maximal contrast when the reference arm cycles through linear states only [12]. Otherwise, single channel PS-OCT is immune to fiber bending artifacts including those of the catheter [2]. In comparison, the dual-channel approach is subject to both the temperature and fiber bending artifacts (most importantly catheter bending artifacts) seen in single-detector PS-OCT, along with additional artifacts generated by slight changes in the alignment of the additional optical components.

To address calibration to prevent artifacts with PS-OCT, examine fossilized teeth as a robust standard, as the process of fossilization ensures the standards will remain unchanged. Here, we chose a prehistoric European horse tooth and prehistoric megalodon shark tooth. We recorded the maximum difference in backreflection intensity as the polarization of incident light was rotated. The process was repeated with bovine meniscus, tendon, and a chemically and mechanically damaged tendon sample, which were correlated with histology.

Using these teeth as a reliable, relatively cheap, robust standard, we found the megalodon tooth had both a moderately high birefringence and low standard error (9.75 dB ± 0.374), and the prehistoric European horse tooth recorded a higher birefringence (10.8 dB ± 1.281). Specifically, we found this fossilized megalodon tooth to represent an excellent standard, with birefringence values between highly birefringent tissues such as tendon and moderately birefringent tissues such as meniscus. While the horse tooth may be slightly more reliable, they are challenging to obtain, unlike the megalodon samples.

In testing these highly in moderately birefringent tissues, we found that tendon demonstrated a high birefringence as anticipated (10.6 dB ± 0.51). In addition, we used tendon which had been both mechanically and chemically degraded to demonstrate the sensitivity of the technique. In contrast to the healthy tendon, this treated sample recorded a lower birefringence value, as expected (4.2 dB ± 0.49). Furthermore, knowing the collagen patterns in the portion of normal meniscus which we imaged, we found the anticipated interweaving. This consisted of organized bundles which were not parallel with the surface. The meniscal birefringence was therefore moderate (3.4 dB ± 0.4). All imaging results were consistent with histologic staining.

These results demonstrate the ease and effectiveness of this clinical standard. By mounting a prehistoric, fossilized tooth within a paraffin mold to prevent movement, and marking the tooth with two dye spots as imaging guides, the standard becomes easily transportable, reproducible, and efficient. Quickly rotating the polarization paddles (or rotating the polarization through another mechanism) while the beam is focused on the marked plane, and comparing the birefringence of the tooth to the preestablished value, allows general calibration of the system. As clinical work based on categorical data relies heavily on ease of use and reliability, this system represents a simple, inexpensive, and much more efficient alternative to the fragile and more complicated typical filter setup for calibration. It allows the physician to judge whether the measurements generated by the system are accurate or altered, which can influence treatment plans. Although an unorthodox approach, we believe this type of standard addresses the problem of reliable and easy calibration, and could be of great use in the clinical setting, as well as with frequent PS-OCT imaging in research labs.
